# Functional disability and health-related quality of life among systemic sclerosis patients in Bangladesh

**DOI:** 10.1186/s41927-022-00291-x

**Published:** 2022-09-30

**Authors:** Sigma Hossain, Minhaj Rahim Choudhury, Md. Mahmudul Haque, Surayea Yeasmin, Farzana Hossain, Mohammad Mostafa Zaman

**Affiliations:** 1 Department of Rheumatology, Bangabandhu Sheikh Mujib Medical University (BSMMU), Shahbag Avenue, Dhaka, Bangladesh; 2 Department of Community Medicine, National Institute of Preventive and Social Medicine (NIPSOM), Dhaka, Bangladesh; 3 National Institute of Preventive and Social Medicine (NIPSOM), Dhaka, Bangladesh; 4 WHO Bangladesh, Dhaka, Bangladesh

**Keywords:** Systemic sclerosis, Health-related quality of life, Functional disability, Short Form 36 (SF-36), Health Assessment Questionnaire-Disability Index (HAQ-DI)

## Abstract

**Objective:**

To assess the relationship between functional disability and health-related quality of life (HRQoL) among systemic sclerosis (SSc) patients.

**Methodology:**

This cross-sectional study was carried out on 78 adults who met the classification criteria for SSc defined by the American College of Rheumatology/European League of Rheumatology (ACR/EULAR)-2013. The Bangla version of Short Form 36 (SF-36) and Health Assessment Questionnaire-Disability Index (HAQ-DI) were used to measure HRQoL and functional disability in SSc patients.

**Results:**

The patients' median [IQR] HAQ-DI was 1.4 [0.6–2.1], with 37.2% having a mild functional disability, 33.3 percent having a moderate functional disability, and 29.5 percent having a severe functional disability. The hygiene and activity domains of the HAQ-DI obtained the highest scores, 2.0 [0.0–3.0] and 2.0 [1.0–3.0], respectively. The Physical Component Summary (PCS) and Mental Component Summary (MCS) of the SF-36 had median [IQR] values of 26.2 [15.0–58.1] and 42.0 [19.6–60.6]. The highest score was 50.0 [25.0–75.0] in social functioning. The PCS of the SF-36 was moderately correlated with the HAQ-DI (r_s_ = − 0.629, *P* < 0.001) and the MCS of the SF-36 was weakly correlated with the HAQ-DI ((r_s_ = − 0.344, *P* < 0.001). Age, female sex, and incomplete fist closure substantially influenced functional status. Calcinosis, Raynaud's Phenomenon, and flexion contracture significantly diminished the quality of life.

**Conclusions:**

Functional disability negatively affects health-related quality of life. Age, Musculoskeletal, and skin involvement are significantly associated with poor quality of life and functional disability. Therefore, treatment strategies should be aimed at reducing functional disability, which will enhance the HRQoL of SSc patients.

## Key messages

What is already known about the subject?

- Functional ability and health-related quality of life are frequently compromised in patients with systemic sclerosis. As a result, they are often functionally disabled and have a poor overall health-related quality of life.

What does this study add?

- A moderate correlation was found between the physical component summary and the HAQ-DI, while the mental component summary weakly correlated with HAQ-DI.
- Functional status was significantly affected by age, female gender, and incomplete fist closure, whereas calcification, Raynaud's phenomenon, and flexion contracture significantly affected the quality of life.

How might this impact clinical practice or future developments?

- Treatment strategies should be focused on improving either of these factors to reduce the functional disability and improve SSc patients' quality of life.

## Introduction

Systemic sclerosis (SSc) is a chronic autoimmune disease. The most prominent feature is the process of progressive fibrosis resulting from the excessive deposition of extracellular matrix components in different tissues and organs [1]. It is mainly divided into two major clinical subtypes- diffuse cutaneous (dc) and limited cutaneous (lc) SSc. In dc involvement, skin thickening occurs proximal to the elbows and knees (upper arms, thighs, anterior chest, abdomen) that is documented at any time during the illness, whereas, in lc involvement, there is either no skin thickening or thickness limited to the distal extremities (never proximal to the elbows or knees) throughout the illness [2]. Globally, its prevalence is estimated between 3 and 24 per 100,000 population and appears to be higher in North America and Australia than in Europe and Japan [3]. However, data are limited regarding the Asian subcontinent.

The definition of disability encompasses various aspects. Illness, impairment, limitation, and handicap are terms directly associated with the concept of disability [4]. SSc is responsible for skin, tendon, joint, and vessel damage, which leads to disability [5]. The hallmark feature of SSc is thickened skin. Moreover, skin manifestations include swollen hands (and sometimes feet), pruritus, telangiectasias, calcinosis, dermal ulcers, digital tip pitting scars, and digital tip gangrene [6]. Raynaud’s Phenomenon (RP), the clinical expression of a disturbance of regulation of cutaneous thermoregulatory vessels [7] is associated with considerable disease-related morbidities such as pain, impaired hand function, and increased reliance on others[8]. Musculoskeletal (MSK) involvement (arthritis/arthralgia, muscle weakness, flexion contracture, etc.) is another factor in the devastating disability in scleroderma patients [9]. These internal and external physical changes lead to severe limitations in work and social activities and psychological distress, ultimately inducing a severe impairment of the health-related quality of life (HRQoL) [5].

Health-related quality of life (HRQoL) is the extent to which one’s usual or expected physical, emotional, and social well-being is affected by a medical condition or its treatment [10]. Due to the rarity and heterogeneity of the disease, not enough is known about the perceived impact of the range of problems faced by individuals living with SSc. Currently, one of the most used generic HRQoL instruments is the Medical Outcomes Short Form-36 (SF-36) [11] which yields two summary scores- the physical component summary (PCS) and mental component summary (MCS). Disability Index of the Health Assessment Questionnaire (HAQ-DI) has been validated and used with people with SSc [12]. It was reported that SF-36 and HAQ-DI complement each other [13]. The PCS of the SF-36 highly correlated with the HAQ, whereas the MCS correlated moderately, which indicated that the QoL of patients with SSc is influenced by functional disability [14]. Moreover, SSc patients' PCS and HAQ-DI scores were adversely affected by joint involvement, ≥ 11 tender points, gastrointestinal involvement, and high skin score [15]. There is no such study among SSc patients in Bangladesh that measures functional disability and identifies the presence and magnitude of physical and mental impairment in these patients. The main aim of this study was to estimate functional disability and HRQoL in patients with SSc and determine the relationship between them. Furthermore, we tried to determine factors that affect functional disability and quality of life.

## Methods

This cross-sectional study initially evaluated 83 (including 78) adult Systemic Sclerosis patients attending the systemic sclerosis clinic at ﻿Bangabandhu Sheikh Mujib Medical University (BSMMU) from September 2017 to October 2018 to assess the relationship between functional disability and health-related quality of life. The study followed the Strengthening the Reporting of Observational Studies in Epidemiology (STROBE) guideline for cross-sectional studies [16]**.**

### Subject enrollment

**Fig. 1:**
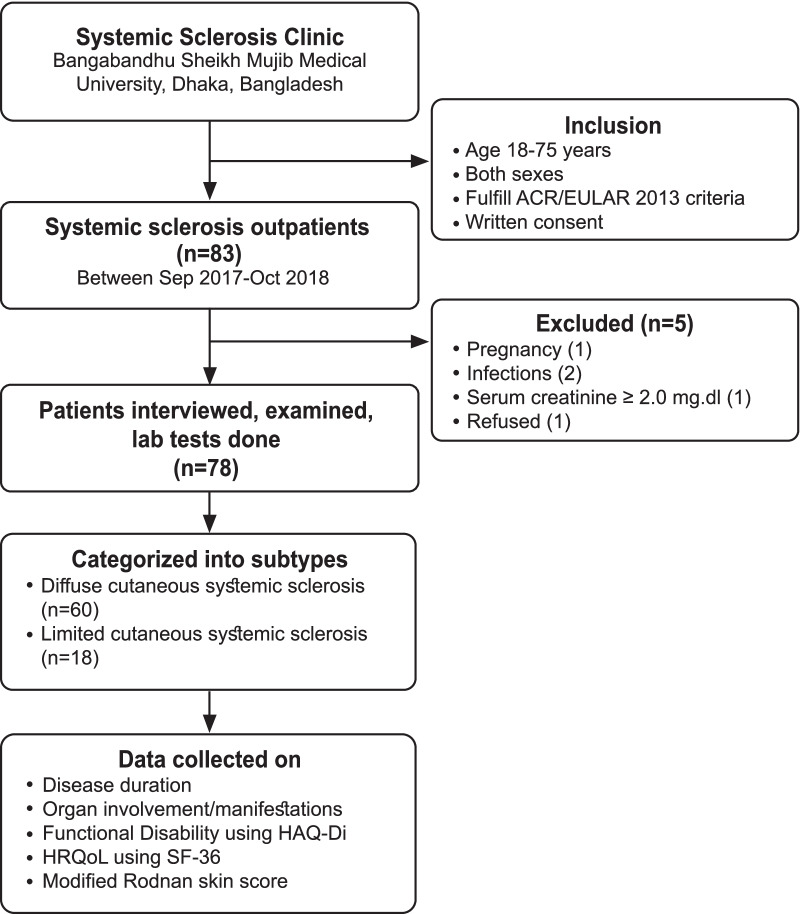
Flow diagram illustrating recruitment of study subject based on the Strengthening the Reporting of Observational Studies in Epidemiology (STROBE) Guidelines for cross-sectional studies. * HAQ-DI—Health Assessment Questionnaire-Disability Index, HRQoL—Health-related quality of life, SF-36—Short Form-36

All systemic sclerosis (SSc)﻿ patients receiving medical care at the Systemic sclerosis clinic in BSMMU were invited to participate in this study as part of their regular follow-up. Of them, 78 consecutive adults (aged 18–75 years) patients of both sexes fulfilled the American College of Rheumatology (ACR)/European League against Rheumatology (EULAR) 2013 classification criteria for SSc [17] with an alpha error of 0.05, a power of 90%, and a correlation coefficient of r = 0.74 [14] were enrolled after giving their informed written consent. Exclusion criteria were the following: Pregnancy, active infections, serum creatinine ≥ 2.0 mg/dl, malignancy, refusal to participate in the study, etc. (Fig. 1). Patients were further categorized into two major clinical subtypes according to skin involvement—diffuse cutaneous (dc) and limited cutaneous (lc) SSc [2]. The duration of dcSSc was categorized as early (< 3 years), intermediate (3–6 years), and late (> 6 years), while the duration of lcSSc was categorized as early (< 5 years), intermediate (5–10 years) and late (> 10 years) [2]. All patients underwent a detailed clinical work-up, including meticulous history taking and thorough physical examination by rheumatologists. Their previous report compiled information on their laboratory tests (ELISA for autoantibodies) and radiological tests (HRCT and echocardiography). In addition, data were collected regarding their disease duration, organ involvement, functional disability using HAQ-DI, health-related quality of life using Short Form-36, and modified Rodnan skin score using a semi-structured questionnaire.

**Organ involvement**

For organ involvement, the following definitions were applied.

### Skin manifestations

Skin thickening and tightening on the fingers was considered Sclerodactyly [18]. In the case of a pinched nose, atrophy of the nasal alae leads to a pinched appearance of the nose. Collagen deposition in the subcutaneous tissue of the facial skin results in the characteristic smooth, taut mask-like face [19]. Calcinosis was characterized by ectopic soft tissue deposition of apatite (calcium hydroxyapatite) [20].

### Vascular manifestations

Raynaud’s Phenomenon was characterized by the digit's biphasic or triphasic cutaneous color change following cold exposure or emotional stress. White/pallor and blue/cyanosis were the two most important colors to make a diagnosis [21]. In addition, loss of substance from digital pulp, with tapering of fingertips, indicated pitting scars of fingertips [22].

### Musculoskeletal manifestations

Arthritis/Arthralgia (Joint tenderness and swelling) were assessed in the wrists, metacarpophalangeal joints, elbows, and knees (8 joints), and the total joint tenderness count and joint swelling count were calculated. The presence of joint involvement was determined by the number of tender joints (≥ 1 tender joint). Fist closure was recorded as the distance from the distal end of the ring finger to the distal palmar crease during maximal fist closure (full closure was scored as zero). Flexion contracture was defined as the sum of bilateral flexion contractures of the wrists, elbows, and knees (6 joints) and palpable tendon friction rubs of the hands, wrists, elbows, knees, ankles, and other areas (6 areas). Muscle involvement was defined by proximal muscle strength ≤ 4/5 [23].

#### Gastrointestinal manifestations

To evaluate Gastrointestinal manifestations, patients were asked for dysphagia and gastroesophageal reflux as an indicator of esophageal involvement, early satiety, vomiting as gastric involvement, diarrhea, constipation, and bloating as intestinal involvement [24]. The patient was considered to have microstomia if the maximal oral aperture was < 40 mm. In addition to the skin's stiffness, the temporomandibular joint's involvement further reduces the oral opening. The oral aperture was measured as the vertical distance from the bottom of the maxillary incisor to the top of the mandibular incisor with the mouth opened using digital calipers. Three consecutive measurements were performed, with a 5 s rest interval, and averages were calculated [25].

### Cardiopulmonary manifestations

Evidence of Interstitial Lung Disease (ILD) (fibrosis and ground glass) on HRCT, supported by a restrictive pattern on pulmonary function tests, was accepted as an interstitial pulmonary disease [26]. Pulmonary arterial hypertension (PAH) is a small pulmonary artery disease that causes a progressive increase in pulmonary vascular resistance, leading to right ventricular failure and death. PAH was considered when there was an elevated mean pulmonary artery pressure (mPAP) of ≥ 25 mm Hg at rest or ≥ 30 mm Hg during exercise with a normal pulmonary capillary wedge pressure (PCWP) of < 15 mm Hg by right heart catheterization [27]. Although right heart catheterization was the gold standard for confirming a diagnosis of PAH, it was not performed on every patient because it was not available in our setting. Furthermore, we did not advise patients to undergo any investigations because the study was cross-sectional. Instead, we reviewed their medical records and gathered data on pulmonary artery systolic pressure (PASP) from their echocardiography (at rest) reports. The most important non-invasive method for detecting pulmonary hypertension is echocardiography at rest, which is recommended for screening patients at risk in several guidelines [28].

A history of congestive heart failure is defined as heart involvement, cardiac arrhythmia requiring medication, pericarditis or moderate-to-large pericardial effusion, cardiomegaly, or a cardiothoracic ratio > 0.5 on the chest radiograph [23].

### Assessment of functional disability

The HAQ-DI is a self-administered 20-question instrument that evaluates functional impairment and contains questions on fine upper-extremity movements, lower-extremity locomotor activity, and activities involving upper and lower extremities [28]. There are eight activity domains (dressing, arising, eating, walking, hygiene, reach, grasp, and typical daily tasks), each with at least two questions, for 20. Patients rate the difficulty they had completing each exercise for each item. Each item had four alternative responses, ranging from 0 (no difficulty) to 3 (extreme difficulty or unable to do all). A mean score ranging from 0 to 3 is calculated for each domain. The total domain scores are divided by the number of domains that responded to get a composite HAQ-DI score. The composite score is given, which ranges from 0 to 3 on an ordinal scale. The HAQ-DI is a scale that ranges from 0.0 (no functional impairment) to 3.0 (severe functional disability), with 0.0–1.0 representing no to mild functional disability, 1.1–2.0 representing moderate functional disability, and 2.1–3.0 representing severe functional disability [28]. This study used the validated Bangla version of this health assessment questionnaire [29].

### Health-related quality of life (HRQoL)

The SF-36 comprises 36 questions grouped into eight domains: physical functioning, role physical, bodily pain, general health, vitality, social functioning, role emotional, and mental health. Each domain is scored separately and ranges from 0 to 100, with 0 indicating the worst HRQoL and 100 indicating the best HRQoL. The domain scores were combined into physical component summary (PCS) and mental component summary (MCS) scores. The validated Bangla version of SF-36 was used [30].

### Modified Rodnan skin score

The Modified Rodnan Skin Score (mRSS) is a useful clinical tool that is used to quantify the severity of skin disease [31]. The mRSS was obtained by clinical palpation of 17 body areas (fingers, hands, forearms, upper arms, chest, abdomen, thighs, lower legs, and feet) and subjective averaging of the thickness at each specific site from 0 (normal) to 3 (very thick). The maximum score is 51 [32].

### Statistical analysis

Data were analyzed with the help of SPSS (Statistical package for social sciences) (26.0 version: IBM Corp., Armonk, NY, USA). Means and standard deviations for continuous variables and frequency distributions for categorical variables were used to describe the characteristics of the total sample. A median with an interquartile range was used to describe the continuous variables in skewed data. Associations of categorical data were assessed using the Chi-square test and Fisher Exact test, while associations of continuous data were evaluated using Student’s t-test. In skewed data regarding HAQ-DI and SF-36, the Mann–Whitney U test was used to determine the association. Variables with p values of < 0.05 during univariate analysis were selected for further multiple linear regression. Three separate models were built for PCS (model 1), MCS (model 2), and HAD-DI (model 3). For model 1, the explanatory variables were sclerodactyly, calcinosis, incomplete fist closure, RP, arthralgia/ arthritis, muscle weakness, and flexion contracture. For model 2, the explanatory variable was calcinosis, while for model 3, the explanatory variables were calcinosis, incomplete fist closure, arthralgia/ arthritis, muscle weakness, and flexion contracture. Every model was adjusted for age and sex. Spearman’s rank coefficient to assess the correlation between two quantitative variables. The Spearman’s coefficient values were interpreted as excellent (> 0.91), good (0.90–0.71), moderate (0.70–0.51), fair (0.50–0.31), or little to none (< 0.30) [33]. A p-value of < 0.05 was considered statistically significant.

### Ethical implications

The study was approved by the Institutional Review Board (IRB) of Bangabandhu Sheikh Mujib Medical University (BSMMU) (BSMMU/2018/5657) and performed according to the Declaration of Helsinki principles. Informed written consent was obtained from all patients before enrolment.

## Results

### Demographic, clinical, and laboratory features of the SSc patients

The mean age of the patients was 36.5 ± 11.3 years, where 71 (91.0%) were female. Sixty patients (76.9%) had dcSSc whereas 18 patients (23.1%) had lcSSc. Out of 60 patients with dcSSc, 25 (41.7%) had 3–6 years, and 18 (30.0%) had more than 6 years, while among 18 patients with lcSSc, 8 (44.4%) patients had this disease for < 5 years. Sclerodactyly was present in 66 (84.6%) patients and 61 (78.2%) had pinch nose and 47 (60.3%) had calcinosis. Raynaud’s Phenomenon was found in 70 (89.7%) patients and pitting scars of fingertips in 53 (67.9%) patients. Thirty-seven (47.4%) patients had arthralgia/ arthritis. Sixty-one patients (78.2%) had microstomia, and 47 (60.3%) had gastroesophageal reflux. The median [IQR] mRSS was 19.0 [10.0–32.0]. Antibody profiles were available for 63 patients. The frequency of antinuclear, anti-topoisomerase and anticentromere antibodies were 37 (58.7%), 24 (38.1%,) and 2 (3.2%), respectively (Table 1).

**Table 1:** Demographic, clinical, and laboratory features of the systemic sclerosis (SSc) patients

Variables	All SSc patients	Diffuse cutaneous systemic sclerosis	Limited cutaneous systemic sclerosis	*P*
	(n = 78)	(n = 60)	(n = 18)	
Age, mean (standard deviation) years	36.5 (11.3)	36.8 (10.3)	35.3 (14.3)	0.69^a^
Female	71 (91.0)	56 (93.3)	15 (83.3)	0.34^b^
Skin manifestations				
Sclerodactyly	66 (84.6)	54 (90.0)	12 (66.7)	0.03^b^
Pinch nose	61 (78.2)	51 (85.0)	10 (55.5)	0.02^b^
Calcinosis	47 (60.3)	42 (70.0)	5 (27.8)	0.00^c^
Vascular manifestations				
Raynaud’s Phenomenon	70 (89.7)	57 (95.0)	13 (72.2)	0.02^b^
Pitting scars of fingertips	53 (67.9)	42 (70.0)	11 (61.1)	0.567^c^
Musculoskeletal manifestations				
Arthritis/arthralgia	37 (47.4)	28 (46.7)	9 (50.0)	0.99^c^
Can’t close fist properly	32 (41.0)	25 (41.7)	7 (38.9)	0.99^c^
Muscle weakness	32 (41.0)	26 (43.3)	6 (33.3)	0.59^c^
Flexion contracture	16 (20.5)	10 (16.7)	6 (33.3)	0.18^b^
Gastrointestinal manifestations				
Microstomia	61 (78.2)	52 (86.7)	9 (50.0)	0.00^b^
Gastro-oesophageal reflux	47 (60.3)	39 (65.0)	8 (44.4)	0.17^c^
Modified Rodnan skin score, Median (Interquartile range)	19.0 (10.0–32.0]	20.0 (15.0–34.0)	10.0 (6.0–21.0)	0.02^d^
Antibody	(n = 63)	(n = 48)	(n = 15)	
Antinuclear antibody	37 (58.7)	26 (43.3)	11 (64.7)	0.17^c^
Anti-topoisomerase antibody	24 (38.1)	20 (33.3)	4 (22.2)	0.41^c^
Anti-centromere antibody	2 (3.2)	2 (3.3)	0 (0.0)	0.99^b^
Echocardiography findings	(n = 24)	(n = 16)	(n = 8)	
Valvulopathy (Tricuspid regurgitation)	24 (48.0)	16 (27.1)	8 (44.4)	0.17^c^
Pulmonary artery systolic pressure				
Normal (12–15 mmHg)	24 (48.0)	21 (55.3)	3 (25.0)	0.07^c^
Increased ((≥ 25 mmHg)	26 (52.0)	17 (44.7)	9 (75.0)	
High resolution CT scan	(n = 17)	(n = 9)	(n = 8)	
Ground glass pattern	10 (58.8)	5 (8.3)	5 (27.8)	0.05^b^
Fibrosis	7 (41.2)	4 (6.7)	3 (16.7)	0.34^b^

a = Student’s *t*-test, b = Fisher’s Exact test, c = Chi-square test, d = Mann–Whitney U test

### HAQ-DI and SF-36 of the SSc patients

The median [IQR] HAQ-DI score was 1.4 [0.6–2.1]. There was no significant statistical difference between the dcSSc and lcSSc regarding the HAQ-DI score. As per the HAQ-DI score, 29 (37. 2%) had a mild functional disability, 26 had moderate (33.3%,) and 23 (29.5%) had a severe functional disability. The median [IQR] PCS and MCS were 26.2 [15.0–58.1] and 42.0 [19.6–60.6] respectively. There was no significant statistical difference between the dcSSc and lcSSc regarding PCS and MCS scores (Table 2).

**Table 2:** Outcome measure scores (HAQ-DI and SF-36) of patients with systemic sclerosis, median [interquartile range]

Scores	All SSc patient (n = 78)	Diffuse cutaneous systemic sclerosis (n = 60)		Limited cutaneous systemic sclerosis (n = 18)	*P*
HAQ-DI scores					
Dressing	1.0 [0.0–2.0]	1.0 [0.0–1.7]	1.0 [0.0–2.0]		0.40^a^
Rising	1.0 [0.0–1.2]	1.0 [0.0–2.0]	1.0 [0.0–1.0]		0.16^a^
Eating	1.0 [0.0–3.0]	1.0 [0.0–3.0]	1.0 [0.0–2.2]		0.25^a^
Walking	1.5 [1.0–2.0]	2.0 [1.0–2.0]	1.0 [0.0–2.0]		0.49^a^
Hygiene	2.0 [0.0–3.0]	2.0 [0.0–3.0]	1.5 [0.0–3.0]		0.91^a^
Reach	1.0 [0.0–3.0]	1.0 [0.2–3.0]	1.0 [0.0–2.0]		0.28^a^
Grip	1.0 [0.0–3.0]	1.0 [0.0–3.0]	2.0 [0.0–3.0]		0.50^a^
Activity	2.0 [1.0–3.0]	2.0 [1.0–3.0]	2.0 [0.0–3.0]		0.97^a^
Total HAQ-DI	1.4 [0.6–2.1]	1.3 [0.5–2.1]	1.4 [0.7–2.0]		0.70^a^
Disability index, n (%)					
Mild	29 (37.2)	22 (36.7)	7 (38.9)		
Moderate	26 (33.3)	19 (31.7)	7 (38.9)		0.72^b^
Severe	23 (29.5)	19 (31.7)	4 (22.2)		
SF-36 scores					
Physical functioning	20.0 [10.0–55.0]	20.0 [10.0–48.7]	15.0 [8.7–66.2]		0.99^a^
Role physical	0.0 [0.0–100.0]	0.0 [0.0–100.0]	0.0 [0.0–81.2]		0.65^a^
Role emotional	0.0 [0.0–100.0]	0.0 [0.0–100.0]	33.0 [0.0–100.0]		0.76^a^
Energy/Fatigue	20.0 [10.0–40.0]	17.5 [10.0–35.0]	25.0 [18.7–46.2]		0.08^a^
Emotional wellbeing	40.0 [20.0–61.0]	40.0 [17.0–60.0]	48.0 [34.0–46.2]		0.17^a^
Social functioning	50.0 [25.0–75.0]	50.0 [25.0–75.0]	56.2 [34.4–78.1]		0.62^a^
Pain	45.0 [22.5–67.5]	45.0 [23.1–67.5]	45.0 [22.5–80.0]		0.88^a^
General health	20.0 [8.7–45.0]	15.0 [5.0–38.7]	30.0 [23.7–61.2]		0.01^a^
Physical component summary	26.2 [15.0–58. 1]	26.2 [15.3–53.1]	31.6 [14.4–60.9]		0.59^a^
Mental component summary	42.0 [19.6–60.6]	37.0 [17.1–59.7]	48.1 [23.3–64.8]		0.28^a^

a = Mann–Whitney U test, b = Chi-square test,

HAQ-DI: Health Assessment Questionnaire-Disability Index, SF-36: Short Form-36

### Correlation between HAQ-DI with PCS and MCS of the SF-36 score

PCS of the SF-36 moderately correlated with the HAQ-DI (r_spearman_ = − 0.648) was statistically significant (*P* < 0.001). In contrast, the MCS of the SF-36 weakly correlated with the HAQ-DI (r_spearman_ = − 0.366, *P* < 0.001) (Table 3).

**Table 3:** Spearman correlation coefficient of domains of SF-36 (Short Form-36) with HAQ-DI (Health Assessment Questionnaire-Disability Index)

SF-36 domains	Spearman correlation with HAQ-DI	*P*
Physical functioning	− 0.644	< 0.00
Role physical	− 0.449	< 0.00
Role emotional	− 0.202	0.08
Energy / Fatigue	− 0.351	0.00
Emotional wellbeing	− 0.366	0.00
Social functioning	− 0.273	0.02
Pain	− 0.493	< 0.00
General health	− 0.342	0.00
Physical component summary	− 0.629	< 0.00
Mental component summary	− 0.344	0.00

### Comparison of HAQ-DI scores and SF-36 scores with different disease duration among SSc patients

No significant difference was observed among patients with different disease duration regarding HAQ-DI scores and SF-36 scores (Table 4).

**Table 4:** Comparison of HAQ-DI score and SF-36 scores among SSc patients with different disease duration (in years), median [interquartile range]

	Early	Intermediate	Late	*P*
DcSSc patients				
HAQ-DI scores	1.2 [1.1–1.9]	0.8 [0.4–2.2]	1.7 [0.7–2.1]	0.448^a^
SF-36 scores				
Physical component summary	40.0 [20.4–55.6]	34.1 [15.0–63.7]	21.9 [15.0–38.1]	0.138^a^
Mental component summary	50.2 [20.0–69.9]	44.3 [17.2–66.3]	28.4 [15.7–57.2]	0.202^a^
LcSSc patients				
HAQ-DI scores	1.3 [0.3–1.9]	1.9 [0.2–2.1]	1.5 [0.8–1.9]	0.957^a^
SF-36 scores				
Physical component summary	24.1 [12.5–64.8]	25.0 [14.4–60.6]	58.1 [24.7–66.6]	0.704^a^
Mental component summary	55.4 [24.2–78.2]	33.6 [27.3–61.2]	47.4 [15.0–64.9]	0.884^a^

For DcSSc, early (< 3 years), intermediate (3–6 years) and late (> 6 years), for LcSSc, early (< 5 years), intermediate (5–10 years) and late (> 10 years)

^a^Mann-Whitney U test,

HAQ-DI: Health Assessment Questionnaire Disability Index; SF-36: Short Form-36, DcSSc: diffuse cutaneous systemic sclerosis, LcSSc: limited cutaneous systemic sclerosis,

### Factors affecting SF-36 and HAQ-DI of the SSc patients

The PCS was affected by sclerodactyly (*P* = 0.046), calcinosis (*P* = 0.043), incomplete fist closure (*P* = 0.015), Raynaud’s phenomenon (*P* = 0.044), arthralgia/ arthritis (*P* = 0.003), muscle weakness (*P* = 0.002), flexion contracture (*P* = 0.005). The MCS was affected by calcinosis (*P* = 0.003). The HAQ-DI was affected by calcinosis (*P* = 0.021), incomplete fist closure ( *P* < 0.001), arthralgia/ arthritis (*P* = 0.020), muscle weakness (*P* = 0.001), flexion contracture (*P* = 0.006) (Table 5). Multiple linear regression analysis found that PCS was significantly affected by Raynaud’s Phenomenon (standardized β = − 0.256) and flexion contracture (standardized β = − 0.257) while MCS was significantly affected by calcinosis (standardized β = − 0.287). Functional status was significantly affected by age (standardized β = − 0.282), female gender (standardized β = − 0.246) and incomplete fist closure (standardized β = − 0.324) (Table 6).

**Table 5:** Physical component summary and mental component summary of SF-36 and HAQ-DI of the patients according to clinical manifestations (n = 78), median [interquartile range]

Clinical manifestations	Physical component summary	Mental component summary	HAQ-DI
Sclerodactyly	24.0 [15.0–54.4]*	33.4 [17.6–61.0]	1.5 [0.7–2.1]
	47.2 [36.2–71.2]	48.5 [35.6–55.9]	1.0 [0.4–1.4]
Calcinosis	21.9 [13.7–53.1]*	23.7 [12.7–57.2]**	1.7 [0.7–2.2] *
	38.7 [21.3–60.9]	51.8 [32.8–70.3]	1.2 [0.5–1.5]
Incomplete fist closure	21.9 [13.9–39.1]*	32.6 [17.6–58.9]	2.0 [1.5–2.2]**
	38.7 [16.2–63.3]	46.4 [20.5–70.3]	0.9 [0.3–1.6]
Raynaud’s Phenomenon	25.0 [15.0–53.1]*	37.0 [18.6–60.6]	1.4 [0.7–2.1] *
	65.3 [21.1–73.7]	51.3 [21.0–77.3]	0.6 [0.2–1.6]
Pitting scar	23.7 [15.0–55.6]	33.2 [16.7–60.6]	1.4 [0.7–2.1]
	39.4 [18.1–59.1]	45.6 [21.9–60.7]	1.2 [0.3–1.8]
Arthralgia/arthritis	21.9 [12.5–43.1]**	33.2 [17.1–51.7]	1.6 [1.1–2.2] *
	39.4 [20.4–64.4]	50.4 [20.3–70.4]	1.1 [0.4–2.1]
Muscle weakness	21.2 [13.3–37.0]**	33.0 [15.8–62.4]	2.0 [1.1–2.2]**
	38.7 [21.2–63.3]	47.8 [21.9–60.6]	1.2 [0.4–1.8]
Flexion contracture	17.9 [9.7–23.3]**	28.9 [15.8–60.3]	2.1 [1.5–2.2]**
	37.2 [17.6–60.0]	45.5 [20.5–63.1]	1.2 [0.4–2.1]
Gastro-oesophageal reflux	23.1 [15.0–50.6]	44.2 [18.0–61.9]	1.5 [0.7–2.1]
	31.2 [21.2–65.0]	39.9 [20.7–60.5]	1.2 [0.4–1.9]
Microstomia	25.0 [15.6–55.6]	44.2 [21.9–60.0]	1.4 [0.6–2.1]
	38.1 [11.9–64.4]	32.7 [16.1–70.4]	1.0 [0.3–1.9]
Pulmonary arterial hypertension	21.8 [13.7–42.8]	28.8 [21.6–57.2]	1.4 [0.2–2.1]
	27.8 [15.3–60.0]	48.7 [13.7–66.3]	1.2 [0.6–1.9]
Female sex	25.0 [15.0–53.1] *	33.6 [18.0–59.5]	1.5 [0.7–2.1] *

All *P* values reached from Mann–Whitney test, ^*^P < 0.05, **P < 0.01, SF-36 indicates Short Form-36; HAQ-DI: Health Assessment Questionnaire-Disability Index

**Table 6:** Predictors for impaired health-related quality of life measured by physical component summary (PCS) and mental component summary (MCS) of SF-36 and functional disability measured by HAQ-DI among systemic sclerosis patients

Variables	Standardized beta coefficients for		
	PCS	MCS	HAQ-DI
Age (in years)	− 0.149	− 0.127	0.282*
Sex (men = 1, women = 2)	− 0.164	− 0.117	0.246*
Sclerodactyly (no = 1, yes = 2)	− 0.013	–	–
Calcinosis (no = 1, yes = 2)	− 0.053	− 0.287*	0.038
Incomplete fist closure (no = 1, yes = 2)	− 0.080	–	0.324*
Raynaud’s Phenomenon (no = 1, yes = 2)	− 0.256*	–	–
Arthralgia/arthritis (no = 1, yes = 2)	− 0.195	–	0.068
Muscle weakness (no = 1, yes = 2)	− 0.120	–	0.165
Flexion contracture (no = 1, yes = 2)	− 0.257*	–	0.191

^*^P < 0.05

R square for model 1 (PCS): 0.351, R square for model 2 (MCS): 0.129, R square for model 3 (HAQ-DI): 0.458

SF-36 indicates Short Form-36; HAQ-DI: Health Assessment Questionnaire-Disability Index

## Discussion

Systemic sclerosis is a rare multisystem autoimmune disease of the connective tissue with the highest case-specific mortality and substantial non-lethal complications [34]. It is characterized by microangiopathy leading to inflammation and fibrosis involving skin and internal organs [35]. Several studies documented that skin manifestations, musculoskeletal manifestations, of organ involvement affect functional disability and quality of life [8, 15, 23, 36–38]. The current study found that age, female gender, and incomplete fist closure significantly affected the functional status. In contrast, calcinosis, Raynaud’s Phenomenon, and flexion contracture significantly affected the quality of life.

Among the 78 patients, the majority had dcSSc, which was consistent with other studies. [14, 36, 39, 40]. However, other studies reported a higher proportion of lcSSc in their studies [38, 41]. Recently, a study among Asian SSc patients found that Asian patients have a distinct genetic susceptibility to systemic sclerosis, earlier systemic sclerosis onset, more diffuse skin involvement, and a more severe clinical phenotype [42]. This dissimilarity of the results might be due to the geo-epidemiological differences in studies.

The frequency of antinuclear, anti-topoisomerase, and anticentromere antibodies was 58.7%, 38.1%, and 3.2%, respectively. A study conducted in Italy found that 100% had antinuclear antibodies, 37.5% had anti-topoisomerase antibodies, and 37.5% had anticentromere antibodies [41]. On the other hand, an Asian study documented 85.5% antinuclear antibody and 22.7% anticentromere antibody-positive patients [42]. The dissimilarity of the result might be because both race and gender significantly influence the occurrence of SSc-related autoantibodies. In addition, multiple genetic and/or environmental factors may also produce specific autoantibodies [43]. Moreover, a study reported that Asian patients have different autoantibody profiles higher frequency of anti-Scl70 and anti-U1-RNP antibodies [42].

The median HAQ-DI was 1.4, which fell in the moderate disability domain. On the other hand, the EUSTAR group [37] and the study of Georges et al. [14]. The mean HAQ-DI among European patients was < 1.0, which fell in the mild disability domain. Several factors might contribute to this dissimilarity of the HAQ-DI score—first, the racial difference—and, secondly, the nature of the study. The present study was cross-sectional with a small sample, whereas those studies were longitudinal with a large sample.

Among the eight domains of SF-36, the social functioning domain had the highest score, while the physical and emotional role domains had the lowest score. A better social functioning domain was also observed in other studies [14, 44]. However, the study of Rosso et al. found that role emotional domain was better than other domains [41]. This might be due to the difference in socio-demographic variation of the study population. The present study found that the MCS score was higher than the PCS score, which indicates that the mental component of SF-36 was better than the physical component, which was consistent with other studies [14, 41, 44, 45]. This might be explained by the fact that SSc patients adapt well to their slowly progressing disease[46] despite significant impairment in physical health. The PCS and MCS scores of dcSSc and lcSSc did not differ significantly, consistent with another study [45].

Functional disability significantly affects the quality of life of SSc patients [39]. Daniele et al. showed that limitations in daily activities were the leading cause of poor quality of life in SSc patients [47]. A moderate but significant negative correlation was found in the present study between the PCS of the SF-36 and the HAQ-DI. However, the study of George et al. reported that PCS was strongly correlated with HAQ-DI. The dissimilarity might be because the present study patients had a moderate physical disability, whereas George et al. had only mild physical disability [14]. Therefore, a weak but significant, negative correlation was present between the HAQ-DI and MCS of the SF-36. Another study found a weak correlation between the HAQ-DI and MCS of the SF-36 [14]. This weak relationship might be due to the psychological adjustment of patients to SSc [13]. Coping with this chronic disabling disease is another reason for such a discrepancy [41].

Several factors were associated with functional disability and poor quality of life. Among these factors, increased age, female gender, musculoskeletal (MSK) involvement, and skin involvement were significantly associated with functional disability and poor quality of life; visceral involvement such as Gastro-oesophageal reflux and pulmonary arterial hypertension (PAH) were found insignificant in this issue. Raynaud’s Phenomenon (RP) is associated with considerable disease-related morbidity across a broad set of domains, including pain, impaired hand function, reduced social participation, body image dissatisfaction, increased reliance on others, and reduced quality of life [8]. We concur with this fact as RP significantly hampers the quality of life of our study patients. Moreover, other skin manifestations such as sclerodactyly and calcinosis are also considerably affected by the HAQ-DI and SF-36 scores. Steen and Medsger's observational study documented that the degree and extent of skin thickening highly correlated with HAQ-DI [36].

Musculoskeletal involvement is a persistent manifestation of patients with SSc and is a significant cause of disability. The most common clinical feature of musculoskeletal involvement is arthralgia; In contrast, the less frequent features are arthritis, flexion contracture, proximal muscle weakness, etc. [48]. The present study's common musculoskeletal manifestations were arthralgia/ arthritis, incomplete fist closure, muscle weakness, and flexion contracture. Arthralgia/ arthritis affected the patients' PCS and MCS of SF-36 and HAQ-DI. Incomplete fist closure, muscle weakness, and flexion contracture significantly affected the patients' PCS component of SF-36 and HAQ-DI. Generalized arthralgia with slight pain and stiffness are SSc's most common articular symptoms on presentation. The course of joint manifestations is either intermittent or chronic remittent. As the cutaneous involvement progresses, an inexorable tethering and contracture of the underlying joints occur with impairment of movement and function [49]. About half of the current study patients had joint pain or swelling, mainly in the upper extremity. This impaired hand function and reduced dexterity and/ or grip significantly affect the functional ability and improve quality of life. Functional difficulties arise because of thickened and bound-down skin resulting in flexion contractures and restricted joint movement [15]. Moreover, reduced thumb abduction contributes substantially to disability in SSc [5]. Weakness can occur based on inflammatory muscle disease. Therefore, reduced quality of life or impairment in function is not unexpected in patients with SSc [15].

Pulmonary arterial hypertension is a frequent and severe pulmonary complication of SSc and has become the leading cause of morbidity and mortality in patients with SSc [50]. However, no significant association was found between PAH and HAQ-DI, consistent with the study of Clement et al. [23]. The HAQ-DI is a targeted musculoskeletal instrument to assess function. Although PAH causes severe disability and death, changes in commonly used measures of PAH severity were not reflected by changes in the HAQ-DI [51]. Increased collagen and other extracellular matrix components in the upper and lower GIT lead to gastrointestinal symptoms in SSc patients. Gastro-oesophageal reflux is one of the leading causes of gastrointestinal discomfort [52]. Several studies reported that gastrointestinal symptoms significantly affect functional disability [36, 37]. However, our patients' gastrointestinal symptoms did not significantly impact their daily activities or quality of life. A higher percentage of patients might explain this discordance with an intermediate and late stage of disease, which helped them cope. Moreover, they followed the lifestyle modification instructions with medications such as domperidone, proton pump inhibitors, etc.

### Strength and limitations

Patients come from around Bangladesh for treatment at BSMMU's systemic sclerosis clinic, the country's only systemic sclerosis clinic. As a result, this study gives an accurate picture of Bangladeshi patients with systemic sclerosis.

There were several limitations to our research. Because of the limited sample size and subgroup analyses, the findings should be taken care of. Only 18 of the 78 individuals had LcSSc, whereas 60 had DcSSc, indicating that group comparisons were not possible. Additional organ assessment was not needed since this was an observational cross-sectional study. Therefore, screening particular organ manifestation findings contain significant missing data and may be somewhat skewed towards patients with more severe organ involvement. Moreover, the laboratory (like autoantibodies test) and radiological tests (e.g., HRCT, echocardiography, etc.) are expensive for most patients.

## Conclusions

From this study, it can be concluded that functional disability negatively affects health-related quality of life in SSc patients in Bangladesh. Furthermore, musculoskeletal and skin involvement is significantly associated with poor quality of life and functional disability. Therefore, treatment strategies should include multi-disciplinary approaches such as physiotherapy, psychotherapy, occupational therapy, social support, and drug therapy. Furthermore, treatment strategies should reduce the functional disability, which will improve the HRQoL of patients with SSc.

## Data Availability

The datasets used and analyzed during the current study are available from the corresponding author on reasonable request.

## Abbreviations

PCS: Physical component summary
MCS: Mental component summary
HRQoL: Health-related quality of life
MSK: Musculoskeletal
HAQ-DI: Health Assessment Questionnaire-Disability Index
DcSSc: Diffuse cutaneous systemic sclerosis
LcSSc: Limited cutaneous systemic sclerosis
mRSS: Modified Rodnan skin score
RP: Raynaud’s Phenomenon
PAH: Pulmonary arterial hypertension
